# Rapeseed Seedling Stand Counting and Seeding Performance Evaluation at Two Early Growth Stages Based on Unmanned Aerial Vehicle Imagery

**DOI:** 10.3389/fpls.2018.01362

**Published:** 2018-09-21

**Authors:** Biquan Zhao, Jian Zhang, Chenghai Yang, Guangsheng Zhou, Youchun Ding, Yeyin Shi, Dongyan Zhang, Jing Xie, Qingxi Liao

**Affiliations:** ^1^College of Resource and Environment, Huazhong Agricultural University, Wuhan, China; ^2^Key Laboratory of Arable Land Conservation (Middle and Lower Reaches of Yangtze River), Ministry of Agriculture, Wuhan, China; ^3^Aerial Application Technology Research Unit, USDA-Agricultural Research Service, College Station, TX, United States; ^4^College of Plant Science and Technology, Huazhong Agricultural University, Wuhan, China; ^5^College of Engineering, Huazhong Agricultural University, Wuhan, China; ^6^Department of Biosystems and Agricultural Engineering, University of Nebraska - Lincoln, Lincoln, NE, United States; ^7^Anhui Engineering Laboratory of Agro-Ecological Big Data, Anhui University, Hefei, China; ^8^College of Science, Huazhong Agricultural University, Wuhan, China

**Keywords:** UAV, remote sensing, vegetation index, rapeseed seedling, stand count, high-throughput phenotyping, agronomic management, seeding performance

## Abstract

The development of unmanned aerial vehicles (UAVs) and image processing algorithms for field-based phenotyping offers a non-invasive and effective technology to obtain plant growth traits such as canopy cover and plant height in fields. Crop seedling stand count in early growth stages is important not only for determining plant emergence, but also for planning other related agronomic practices. The main objective of this research was to develop practical and rapid remote sensing methods for early growth stage stand counting to evaluate mechanically seeded rapeseed (Brassica napus L.) seedlings. Rapeseed was seeded in a field by three different seeding devices. A digital single-lens reflex camera was installed on an UAV platform to capture ultrahigh resolution RGB images at two growth stages when most rapeseed plants had at least two leaves. Rapeseed plant objects were segmented from images of vegetation indices using typical Otsu thresholding method. After segmentation, shape features such as area, length-width ratio and elliptic fit were extracted from the segmented rapeseed plant objects to establish regression models of seedling stand count. Three row characteristics (the coefficient of variation of row spacing uniformity, the error rate of the row spacing and the coefficient of variation of seedling uniformity) were further calculated for seeding performance evaluation after crop row detection. Results demonstrated that shape features had strong correlations with ground-measured seedling stand count. The regression models achieved R-squared values of 0.845 and 0.867, respectively, for the two growth stages. The mean absolute errors of total stand count were 9.79 and 5.11% for the two respective stages. A single model over these two stages had an R-squared value of 0.846, and the total number of rapeseed plants was also accurately estimated with an average relative error of 6.83%. Moreover, the calculated row characteristics were demonstrated to be useful in recognizing areas of failed germination possibly resulted from skipped or ineffective planting. In summary, this study developed practical UAV-based remote sensing methods and demonstrated the feasibility of using the methods for rapeseed seedling stand counting and mechanical seeding performance evaluation at early growth stages.

## Introduction

Unmanned aerial vehicles (UAVs) have become a popular and promising platform for field-based phenotyping (FBP) (Zhang and Kovacs, 2012; Sankaran et al., 2015; Yang et al., 2017). UAVs have many advantages, including flexibility to be quickly deployed, low-attitude imaging and non-invasive observation with ultrahigh spatial resolution, and frequent data collection. They offer great opportunities for field-based high-throughput phenotyping (Araus and Cairns, 2014; Sankaran et al., 2015; Holman et al., 2016). In contrast to the high-throughput phenotyping platforms (HTPPs) in greenhouses or growth chambers, UAV-based HTPPs can obtain detailed information in fields (Yang et al., 2017). Compared with other field-based HTPPs (e.g., unmanned ground vehicle), they provide more effective and simultaneous measurements of all plots in a relative large field (Holman et al., 2016; Liu et al., 2017b). Thus, the emerging UAV-based HTPPs have been increasingly used to evaluate plant water stress (Sullivan et al., 2007; Baluja et al., 2012; Gonzalez-Dugo et al., 2013; Ludovisi et al., 2017), nitrogen content (Kefauver et al., 2017; Krienke et al., 2017), and growth parameters (Brede et al., 2017; Jin et al., 2017; Yue et al., 2017) at field scale. During the FBP of a crop's entire growth period, there is a strong interest in evaluating its growth traits such as canopy cover (Irmak et al., 2000; Breckenridge et al., 2011; Córcoles et al., 2013) and plant height (Bendig et al., 2015; Holman et al., 2016; Schirrmann et al., 2017; Watanabe et al., 2017; Yue et al., 2017). Nevertheless, seedling stand count in early growth stage has not received enough attention, though it is one of the most important traits for crop cultivation and management (Severini et al., 2011; Sankaran et al., 2015; Gnädinger and Schmidhalter, 2017; Liu et al., 2017b).

Seedling stand count is not only critical for determining plant emergence (Jin et al., 2017), density (Liu et al., 2017b) and yield (Zheng et al., 2016) in breeding programs, but also important for other related agronomic practices. For example, plant density derived from seedling stand count is considered as one of the first variables commonly measured in agronomical trials (Liu et al., 2017a). Furthermore, most crops are sown in rows by seeding devices nowadays. The uniformity of the seedling distribution based on seedling stand count can be useful for improving seeding equipment technology. However, traditional manual methods to count the number of seedlings are time-consuming and prone to human errors (Jin et al., 2017; Liu et al., 2017b).

The fast development of field-based high-throughput phenotyping provides new ways to overcome this deficiency (Araus and Cairns, 2014; Chapman et al., 2014; Shi et al., 2016). Shi et al. used a LiDAR system to achieve maize plant locating and counting at mid-growth stages. A laser scanner was used to count maize plants from the side-view (Shi et al., 2013). The mean total errors in plant counting at two different growth stages were, respectively, 24.0 and 10.0%. In addition, weed control and sensing height would impact on the counting accuracy when using a ground-based platform for maize counting at the mid-growth stages. In contrast, Gnädinger et al. carried out maize plant recognition using digital counts in images that captured by an UAV platform at different growth stages (Gnädinger and Schmidhalter, 2017). The authors found that ground cover calculated from segmentation of green areas indicated little correlation (*R*^2^ = 0.023) with plant numbers recorded manually in a field. After image processing via enhancing color contrasts and creating a threshold, a strong correlation (*R*^2^ = 0.89) between digital counts and plant numbers was achieved. These results demonstrated the potential of using field-based HTPPs to achieve crop seedling counting. However, these methods for maize stand counting can hardly be applied to other small crops (Liu et al., 2017a), such as wheat and rapeseed. Maize plants are bigger, with larger plant spacing and more uniform distribution (Jin et al., 2017; Liu et al., 2017b). Instead, some crops have complex leaf overlap with small and variable spacing (Jin et al., 2017), making it difficult to employ these methods directly.

In spite of a number of difficulties, several studies on wheat density estimation have been conducted (Liu et al., 2016, 2017a,b; Jin et al., 2017). The seedling counting of wheat first started with the extraction of features of segmentation objects. Objects were separated from images of vegetation indices (VIs) derived from ultrahigh resolution images. Wheat density was further computed from the objects in conjunction with crop row detection. Liu et al. used a neural network to estimate the number of seedlings in wheat objects using object features (Liu et al., 2017b). The method was applied at three experimental sites with different types and numbers of features. The experimental result showed the estimated wheat density accuracy with an average relative root-mean-square-error (RMSE) of 12.15% for the three experiments. This study demonstrated that extracted feature type and number affected the estimated result. Jin et al. used a supervised classification method to estimate wheat seedling count (Jin et al., 2017). The results also demonstrated that spatial resolution better than 0.40 cm/pixel would improve seedling count estimation. It was possible to retrieve the number of plants per segmentation object through separating the overlapping leaves at this resolution. These efforts for wheat seedling counting have been implemented with the advances in object-based image analysis (OBIA) techniques (Pe-a-Barragán et al., 2011; Torres-Sánchez et al., 2015) and the usage of ultrahigh resolution images collected from field-based HTPPs (Ballesteros et al., 2014; Matese et al., 2015). FBP with high resolution images offers a new means to estimate crop seedling count with sufficient accuracy. Image resolution, observation growth stage, and estimation methods all influence the performance. These results will be beneficial to the study of rapeseed, a crop that also has complex leaf overlap and small and variable spacing, like wheat.

China is one of the main countries for rapeseed production (Fu et al., 2001; Wang et al., 2007; Yu et al., 2014). Mechanical direct-seeding of rapeseed is a method encouraged and supported by the government for agricultural production. Nonetheless, little work has been done on the use of UAV-based technology for rapeseed seedling stand counting. Meanwhile, rapeseed has small seed size with a diameter of 1.9 mm, which can be easily damaged or gathered to block the nozzle of the sowing device to cause poor seeding performance (Yu et al., 2014). Thus, there is an urgent need for field-based seeding performance evaluation to improve agronomy and mechanical seeding technology. As mentioned above, the advances in crop seedling counting will facilitate the development of techniques for rapeseed stand counting. Therefore, the objectives of this study were to (1) use ultrahigh resolution UAV imagery to identify rapeseed seedling objects at two different growth stages; (2) develop multiple regression models for seedling stand counting over these stages; (3) evaluate mechanical seeding performance based on seedling stand count.

## Materials and methods

### Study area and field experiment

This study was conducted in a field plot of 18 × 50 m (central coordinate: 114°21′17.5″E, 30°28′4.2″N) in Wuhan, Hubei province, China in autumn 2016. Rapeseed was seeded in the study area with three different mechanical seeding devices, including a precision pneumatic cylinder-type centralized seeding device (PPCCSD), a rotating disc-type seeding device (RDSD) and a centrifugal metering device (CMD). According to the field experiment, six sample plots were randomly delimited for each seeding device. A total of 18 sample plots were selected for the rapeseed seedling counting survey and estimation. The seedlings in each sample plot were manually counted and recorded after image collection on the same day. Detailed seeding information with the three seeding devices is given in Table 1. Since the difference in the number of rows, these three treatments didn't have the same design density, but had the same ratio of seeding rate to number of rows, 4.5/6 = 6/8 = 0.75, to ensure the consistency of sowing.

**Table 1:** Seeding information for three seeding devices.

**Seeding device**	**Sowing date**	**Seeding rate (kg/ha)**	**Number of rows (row)**	**Theoretical row spacing (cm)**	**Size of subsample(m × m)**	**Sampling sequence**
PPCCSD	07 October 2016	4.5	6	25.0	1.6 × 2.0	1–6
RDSD	09 October 2016	6.0	8	20.0	2.0 × 2.5	7–12
CMD	09 October 2016	6.0	8	20.0	2.0 × 2.5	13–18

**Figure 1:**
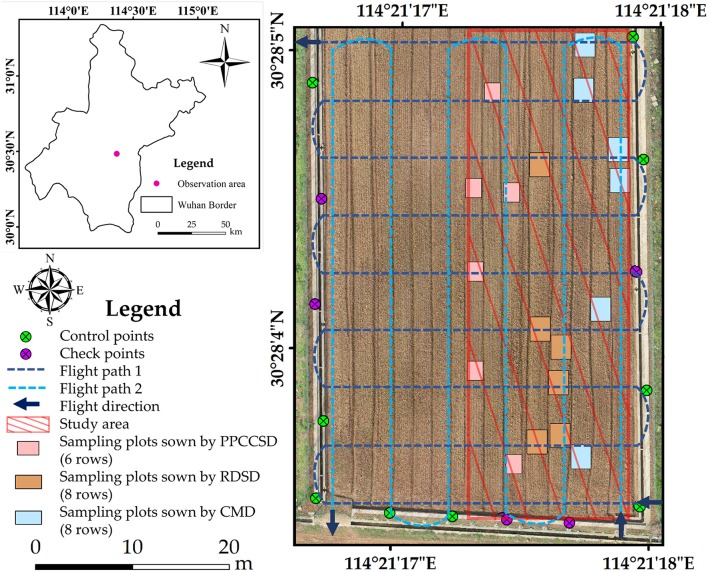
Study area and GCP distribution (the image was captured on 02 November 2016, projected coordinate system was WGS 1984, UTM Zone 50N, and the RMSE in the X and Y directions calculated by check points was 0.76 and 0.74 cm, respectively).

Ground control points (GCPs) were collected by a global navigation satellite system real-time kinematic (GNSS RTK) instrument (UniStrong Science & Technology Co., Ltd, Beijing, China) after image acquisition. There were five check points among a total of 14 GCPs. The study area and the GCP distribution are shown in Figure 1.

### The UAV platform and camera configuration

A Matrice 600 UAV developed by DJI-Innovations (DJI, Shenzhen, China) was used for this study. With the maximum payload of 6.0 kg, its hovering time is close to 16.0 min. This UAV can resist the maximum wind speed of 8.0 m/s and its maximum speed is 18.0 m/s in a windless environment. In this study, the UAV was flying at about 3.0 m/s at an altitude of 20.0 m. The camera was looking with 15° zenith angle. The camera was setting as being parallel to the main flight path during flighting, with a forward overlap of 80.0% and a side overlap of 70.0%. Moreover, two flight plans were specifically designed for the UAV as shown in Figure 1. The two flight paths were perpendicular to each other and one of the path was parallel to the row direction. This study used the configuration to ensure that all the rapeseed plants were covered and imaged (to maximize the cross section viewed of the rapeseed plants). The ortho-mosaic imagery (e.g., Figure 1) was further generated by Pix4D software. The trajectory was automatically controlled by the integrated global position system (GPS) in the UAV with a horizontal accuracy of 1.5 m and a vertical accuracy of 0.5 m.

A digital single-lens reflex Nikon D800 camera (Nikon, Inc, Tokyo, Japan) was installed on the UAV. The camera employed a complementary metal-oxide-semiconductor (CMOS) sensor of 35.9 × 24.0 mm and was equipped with a Nikon 50.0 mm f/1.4 D focal lens to acquire RGB images with 36.3 million effective pixels. The camera was also equipped with a GPS device and a wireless trigger. The GPS information in the images could improve the accuracy of the mosaicked image. Images were captured every 1.0 s automatically during the UAV flight. The captured 24-bit JPEG images with 7,360 × 4,912 pixels were stored on a SD memory card.

The images were collected on 02 and 12 November 2016 at about 11 am local time under clear and calm weather condition. The imaging dates were appropriate because most of the rapeseed seedlings had emerged and were at the growth stage with at least two leaves larger than 1.0 cm^2^. In the study, the 230 images collected on 02 November 2016 were named Stage 1 and the 219 images on 12 November 2016 were named Stage 2.

### Data processing and statistical analysis

**Figure 2:**
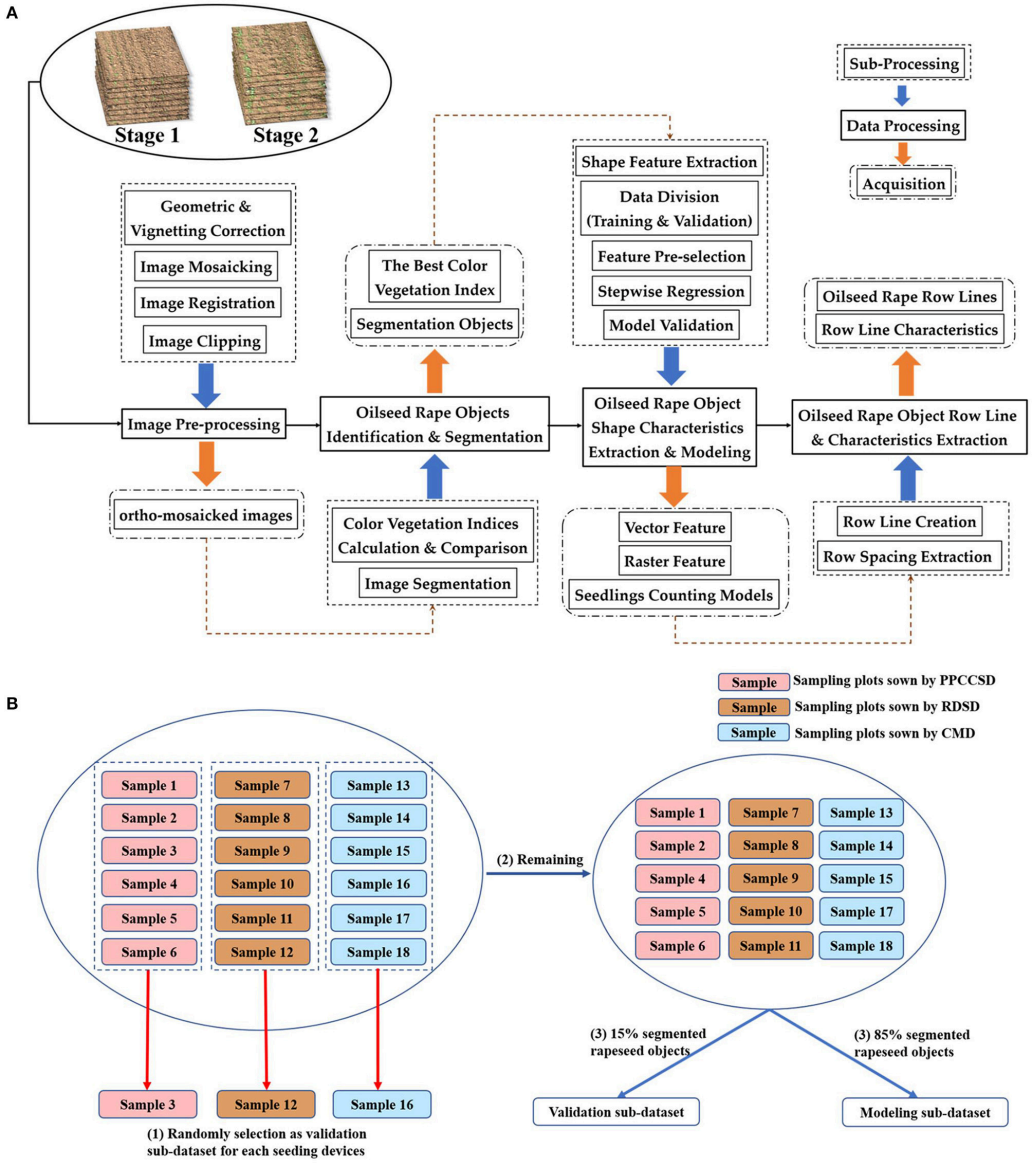
Data processing and analysis flowchart in **(A)**, and data grouping flowchart in **(B)**.

The framework of data processing is illustrated in Figure 2A. The first step was image pre-processing, which included distortion correction of each image, image mosaicking, registration and clipping. The second step was rapeseed object identification and segmentation. These two steps were based on the 18 sample plots, meaning that the processing domain was at image level. Afterwards, some types of shape characteristics for rapeseed objects were extracted for seedling stand count modeling. Finally, seeding performance was evaluated according to the seedling stand count estimation, including crop row detection and row characteristic calculation.

#### Image pre-processing

Vignetting and geometric distortion of images were corrected by the free software, Capture NX-D 1.2.1 (Nikon, Inc, Tokyo, Japan), provided with the camera. Pix4DMapper software (Pix4D, Inc., Lausanne, Switzerland) was used to mosaic the calibrated images, add the GCPs, and calculate RMSE with the check points. The spatial resolution, RMSE X, and RMSE Y for the mosaicked images are shown in Table 2. The mosaicked images were registered and divided into 18 subsets by ERDAS Imagine 2014 (Intergraph Corporation, Madison, AL, USA). In this study, the mosaicked image for Stage 2 was registered to the mosaicked image for Stage 1 with a RMSE of 0.384 pixels. These 18 subsets corresponded to the 18 sample plots according to its sampling sequence (Table 1).

**Table 2:** Information on mosaicked images for two dates.

**Dataset**	**Number of images**	**Acquisition date**	**Flight height (AGL: m)^a^**	**Spatial resolution(cm)**	**GCP/Check point**	**RMSE X (cm)**	**RMSE Y (cm)**
Stage 1	230	02 November 2016	20	0.18	14 / 5	0.76	0.74
Stage 2	219	12 November 2016	20	0.18	14 / 5	0.49	0.75

a *The abbreviation AGL stands for above ground level*.

#### Rapeseed object identification and segmentation

The key to object identification and segmentation was to separate the rapeseed plant objects from the background. Color vegetation indices are widely used for crop identification in agriculture (Xue and Su, 2017). Table 3 lists some common VIs based on visible bands from traditional digital cameras. Since rapeseed is sensitive to the green light, excess green (ExG) (Woebbecke et al., 1995), excess green minus excess red (ExG-ExR) (Meyer and Neto, 2008), normalized green minus red difference index (NGRDI) (Gitelson et al., 2002) and green leaf index (GLI) (Louhaichi et al., 2001) were chosen for rapeseed object identification.

**Table 3:** Common color vegetation indices based on RGB images.

**Color vegetation indices**	**Abbreviation**	**Formula**
Excess green index	ExG	2*G-R-B* (Woebbecke et al., 1995)
Excess red index	ExR	1.4*R-G* (Meyer et al., 1999)
Excess green minus excess red index	ExG-ExR	3*G-*2.4*R-B* (Meyer and Neto, 2008)
Normalized green minus red difference index	NGRDI	*(G-R)/(G+R)* (Gitelson et al., 2002)
Green leaf index	GLI	(2*G-R-B*)/(2*G+R+B*) (Louhaichi et al., 2001)

*R, G, and B represent the digital numbers (DNs) of the color image channels red (R), green (G), and blue (B), respectively*.

Otsu thresholding method was used to separate the rapeseed objects from soil background because of its advantages of quick operation (Vala and Baxi, 2013) and low probability of incorrect segmentation. There are some improved Otsu thresholding methods, but this study chose the typical method because it was the most fundamental segmentation algorithm. The feasibility of using color index-based with Otsu thresholding for segmentation to separate green crop from bare soil has been reported (Meyer and Neto, 2008; Hamuda et al., 2016). In this study, the field scene was relatively simple, with brown bare soil and green rapeseed plants. Color-based Otsu thresholding could achieve an optimal threshold to sperate background and target in this study. The thresholds for the 18 sample plots could be rapidly and automatically obtained.

Precision, Recall and F-measure were used in this study to determine the segmentation effect and accuracy. Overall accuracy and kappa value based on the confusion matrix were also calculated for segmentation evaluation. Precision and Recall are the most basic indicators to reveal the final segmentation results (Xiong et al., 2017). F-measure is an overall factor to balance these two indicators. Precision, Recall and F-measure are defined in terms of true positive (TP), false positive (FP) and false negative (FN) as follows:

(1) $$\begin{array}{l} \text{Precision }=\;\frac{\text{TP}}{\text{TP}+\text{FP}} \end{array}$$

(2) $$\begin{array}{l} \text{Recall }=\;\frac{\text{TP}}{\text{TP}+\text{FN}} \end{array}$$

(3) $$\begin{array}{l} \text{F}-\text{measure }=\;2\times \;\frac{\text{Precision }\times \text{Recall}}{\text{Precision}+\text{Recall}} \end{array}$$

A TP means that the extracted pixel representing rapeseed is indeed rapeseed in the reference. If the extracted pixel does not represent rapeseed, but the reference indicates rapeseed, then it is counted as a FN. For an FP, the extracted pixel represents rapeseed, but the reference doesn't indicate rapeseed.

**Figure 3:**
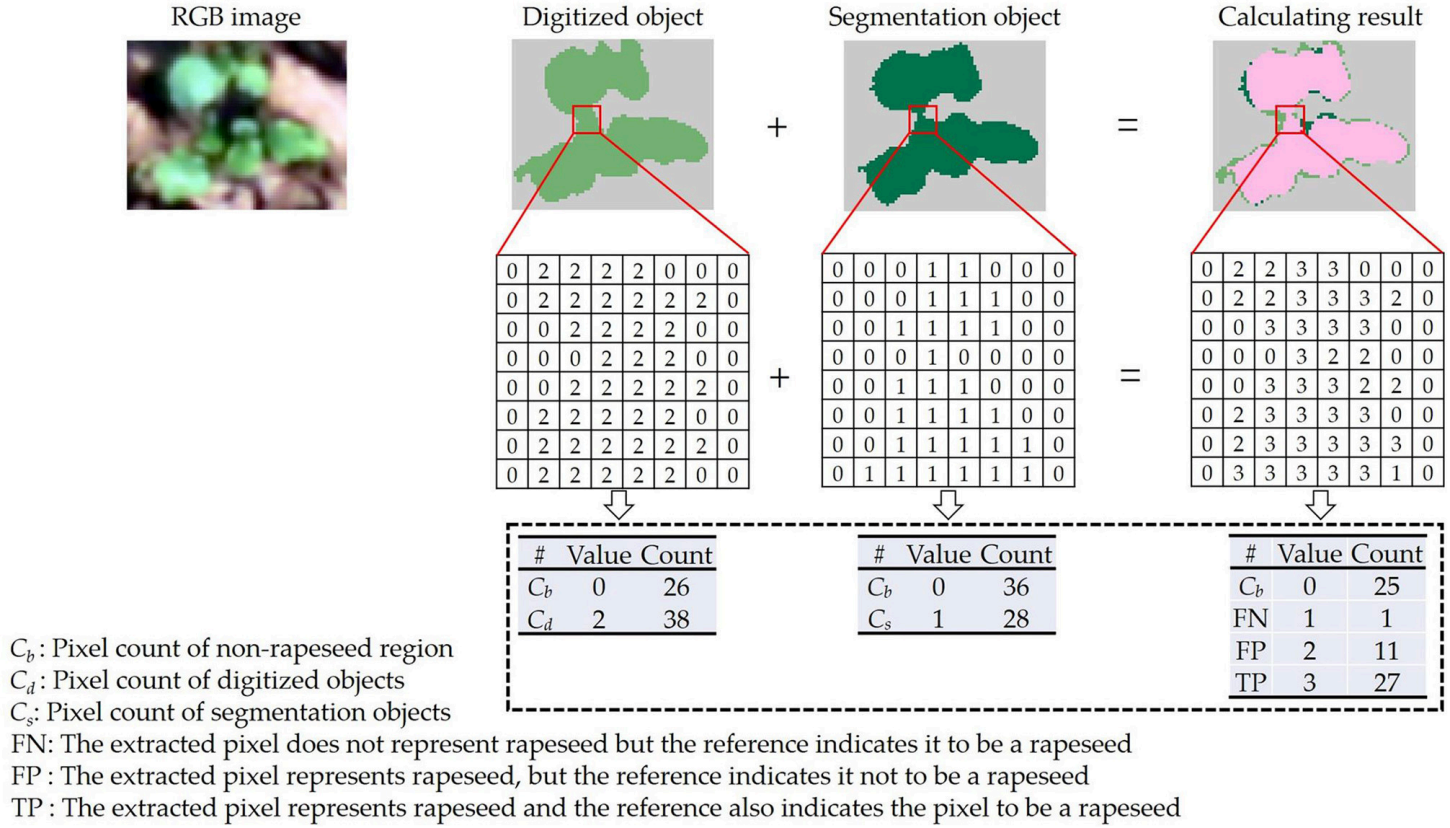
Accuracy assessment of the segmentation object.

In this study, two sample plots were randomly chosen from the two datasets for the segmentation test. Their VI images were validated with a reference image digitized manually in ArcMap 10.3 (ESRI, Redlands, CA). An example was shown in Figure 3. The pixel value of the reference objects was assigned to 2. Meanwhile, the pixel value of the segmentation objects derived from the VI image was assigned to 1. The value of the non-rapeseed region was 0 in all images. Summing the digitized object layer to the segmentation object layer, a new layer with four values (0, 1, 2, and 3) was generated (calculating result in Figure 3). In the new layer, the value of 0 indicated the non-rapeseed region. If a pixel had the value of 1 in the new layer, the pixel only appeared in the segmentation object layer (FN). Similarly, if a pixel had the value of 2, the pixel only appeared in the digitized object layer (FP). The value of 3 indicated that the pixel matched between the digitized and segmentation object layers (TP).

During rapeseed identification and segmentation, the confusion of weeds with rapeseed seedlings was unavoidable. According to the rapeseed growth stages, the possible weeds were eliminated by removing segmentation objects with an area less than 1.16 cm^2^ (about 36 pixels) in this study. Another confusion was due to the disconnected fragment objects after segmentation. They might belong to the same rapeseed plant but were disconnected during image segmentation because of the effect of illumination, imaging angle and motion blur. In this study, these potential disconnected objects were merged if they were no more than 1.0 cm apart.

#### Rapeseed object shape feature extraction and seedling stand count modeling

After the rapeseed object identification and segmentation, the processing domain changed from image level to image object level. Although it was difficult to separate a rapeseed object to independent plant because of the complex overlap, the number of seedlings contained in the object would greatly influence its morphological parameters. For instance, an object with more rapeseed seedlings contains more pixels than an object with a single rapeseed plant. Therefore, there exist differences in shape features between the two objects. This was the base of using shape features to estimate rapeseed seedling stand count. Two types of shape features were calculated after seedling identification and segmentation: vector features and raster features. For vector features, four geometric features were calculated by the minimum enclosing rectangle of an object. Its perimeter (cm) and area (cm^2^) were calculated first and then the length-width ratio and the area-perimeter ratio were calculated. The raster features consisted of 11 shape characteristics, including area count (pixel), perimeter count (pixel), length-width ratio of raster number, border index, shape index, the distribution density of the pixel feature, asymmetry, compactness, roundness, rectangular fit, and elliptic fit (Trimble, 2014). They were calculated by eCognition Developer software (version 8.9, 64-bit, Trimble Germany GmbH, Germany). Table 4 lists these shape features extracted for each of the connected object.

**Table 4:** Fifteen shape characteristics extracted for each connected object (Trimble, 2014).

**Feature**	**Name**	**Meaning**	**Unit**
*F*_1_	Perimeter^a^	The perimeter of an image object's minimum enclosing rectangle	cm
*F*_2_	Area^a^	The area of an image object's minimum enclosing rectangle	cm^2^
*F*_3_	Area-perimeter ratio^a^	The area-to-perimeter ratio of an image object's minimum enclosing rectangle	scalar
*F*_4_	Length-width ratio^a^	The length-to-width ratio of an image object's minimum enclosing rectangle	scalar
*F*_5_	Border index^b^	How jagged an image object is; the more jagged, the higher its border index	scalar
*F*_6_	Area count^b^	The number of pixels forming an image object	pixel
*F*_7_	Roundness^b^	How similar an image object is to an ellipse	scalar
*F*_8_	Compactness^b^	How compact an image object is	scalar
*F*_9_	Shape index^b^	The smoothness of an image object border	scalar
*F*_10_	Length/width^b^	The length-to-width ratio of an image object	scalar
*F*_11_	Rectangular fit^b^	How well an image object fits into a rectangle of similar size and proportions	scalar
*F*_12_	Density^b^	The distribution in space of the pixels of an image object	scalar
*F*_13_	Elliptic fit^b^	How well an image object fits into an ellipse of similar size and proportions	scalar
*F*_14_	Asymmetry^b^	The relative length of an image object, compared to a regular polygon	scalar
*F*_15_	Border length^b^	The sum of pixels along an image object edge	pixel

a Vector feature type and

b *Raster feature type*.

After rapeseed object segmentation and shape feature extraction, the segmented objects in the 18 sample plots for each of the two imaging dates (Stages 1 and 2) were divided into a training dataset and a validation dataset before modeling. To analyze the model performance on each seeding device, one sample plot from the six sample plots for each seeding device was randomly chosen as a validation area, resulting in three validation sub-datasets for the three seeding devices. The rapeseed objects in these three validation sample plots were not used for modeling. Furthermore, 85% of the objects in the remaining 15 sample plots for each imaging date were randomly assigned as the training dataset for modeling, and the other 15% of the objects were used as the fourth validation sub-dataset. Thus, there were four validation sub-datasets and one training dataset as shown in Figure 2B.

It is known that when the absolute linear correlation coefficient is high (i.e., over 0.8) (Riordan and Rundel, 2014; Sanjerehei and Rundel, 2018), the two variables are significant collinear. Accordingly, a pre-selection was conducted for the 15 shape characteristics. Let *a* and *b* be any two shape features and *y* be the ground-measured rapeseed seedling stand count contained in objects. If these two features (*a* and *b*) were significant collinear (|*r*_*ab*_| > 0.8), the feature that had a higher absolute linear correlation coefficient with y would be selected. Otherwise, both features would be the selected characteristics.

In multivariate statistics, it is not sufficient to just evaluate the simple correlation coefficient. Multiple stepwise regression should be evaluated to eliminate the redundant shape features and identify the significant features for multivariate modeling. Consequently, multiple stepwise regression was used for establishing the relationship between object shape characteristics and rapeseed seedling stand count in the segmented rapeseed objects in this study. The statistical analysis was implemented in IBM SPSS Statistics software (version 23, 64-bit, IBM Corporation, USA). The significant levels of variable selection and elimination were 0.05 and 0.10, respectively, which were default settings in SPSS. The validation dataset was used for comparing the estimated stand count values to ground-measured count values. The coefficient of determination (*R*^2^) and RMSE were used as important indicators. Meanwhile, the sum of the estimated rapeseed seedling stand counts for the objects in a sample plot was introduced because the total stand count estimation in each sample plot was the focus in the study. Estimated count sum was rounded to integers. The error rate of the sum (*E*_*s*_) and mean-absolute-error (MAE) among the validation subsets was further used for validation.

(4) $$\begin{array}{l} \text{Estimated count sum }=\;\overset{M}{\underset{i\;=\;1}{\sum }}{ŷ}_{i} \end{array}$$

(5) $$\begin{array}{l} E_{s}\;=\;\frac{\left(\text{Estimated count sum}-\text{Measured count sum}\right)}{\text{Measured count sum}} \end{array}$$

(6) $$\begin{array}{l} \text{MAE }=\;\frac{1}{N}\overset{N}{\underset{i\;=\;1}{\sum }}|{\left(E_{s}\right)}_{i}| \end{array}$$

where *M* is the number of the segmented rapeseed objects in a sample, ŷ is the estimated rapeseed seedling stand count in a segmented rapeseed object, *N* is the number of the validation subsets in a dataset, and |(_*E*_*s*_)*i*_| is the absolute *E*_*s*_ of the *ith* validation subset.

#### Seeding performance evaluation

**Figure 4:**
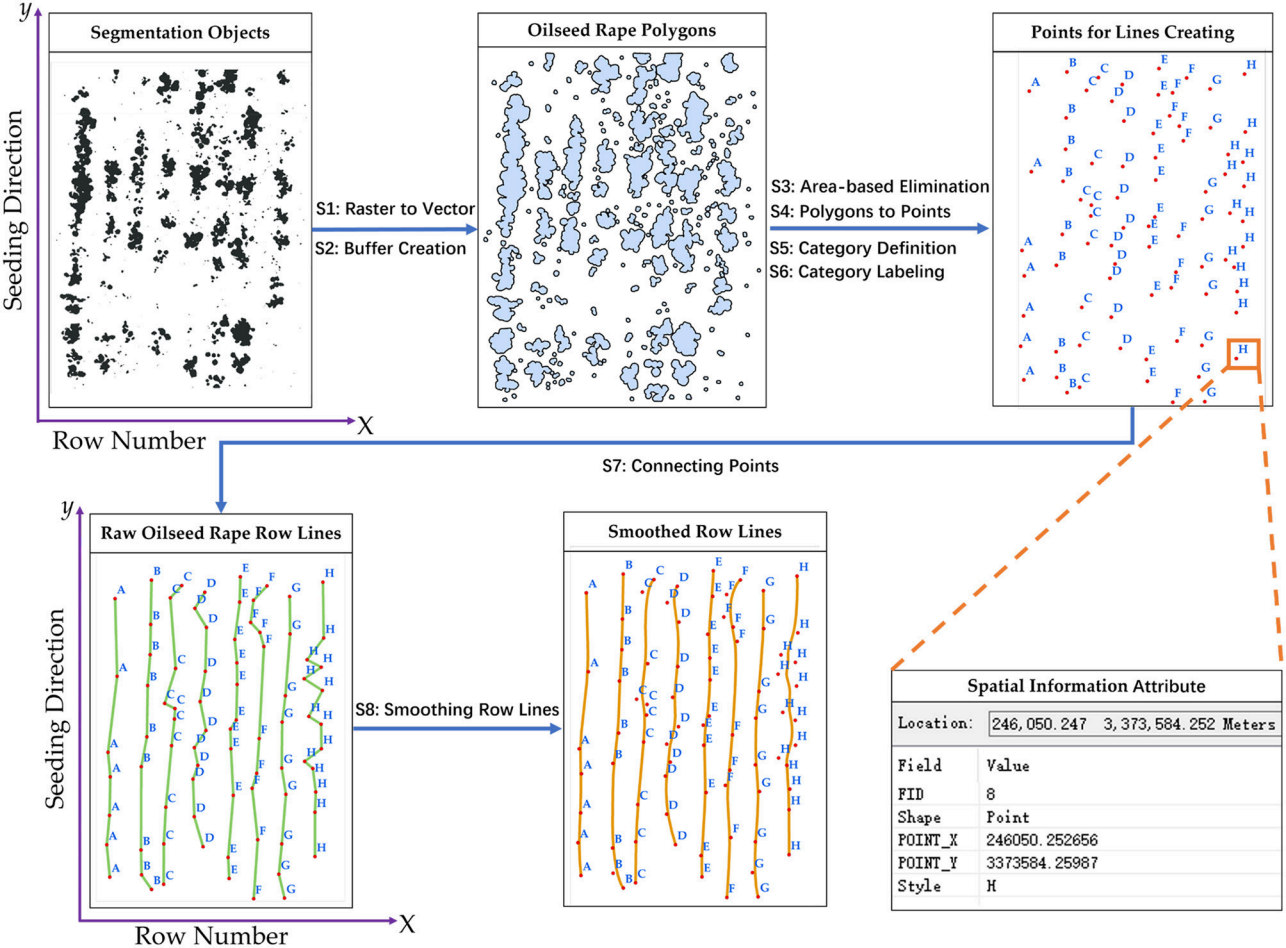
Flowchart for creating rapeseed row lines: S1-raster to vector, S2-buffer creation, S3-area-based elimination, S4-polygons to points, S5-category definition, S6-category labeling, S7- connecting points, and S8-smoothing row lines.

The seeding performance evaluation included two main processes, rapeseed row detection, and row characteristic calculation. In this study, the rapeseed row lines were created by points that were transformed from the segmentation objects and spatial information. The key to row line detection was to clearly define the points a rapeseed row line contained. For this purpose, the points converted from the segmentation objects were categorized and labeled according to the rapeseed row number and the x-axis. The rapeseed row lines were generated by connecting the points with the same labels along the y-axis (seeding direction). Figure 4 shows the flow for creating rapeseed object row lines. It should be mentioned that the area-based elimination (S3) used in Figure 4 was based on a threshold that was the area-median of the segmentation objects in the sample. In fact, the seeding device might be shaking during seeding due to the change of topography or other factors, which could cause irregular seed placement and emergence. The area-based elimination can retain the major rapeseed objects for the row line creation. These processing techniques were written as a Python script and implemented in ArcMap 10.3 (Zandbergen, 2013; ArcGIS, 2014). The validation datasets representing the three different seeding devices were used for calculating row characteristics.

Three row characteristics were calculated, including the coefficient of variation of row spacing uniformity (*CV*_*rs*_), the error rate of the row spacing (*E*_*rs*_), and the coefficient of variation of seedling uniformity (*CV*_*su*_). This study firstly measured the row spacing with the spatial information of any two adjacent row lines. Accordingly, *CV*_*rs*_ and *E*_*rs*_ were calculated by the following formulas:

(7) $$\begin{array}{l} {\bar{X}}_{rs}=\frac{1}{N-1}\overset{N-1}{\underset{i\;=\;1}{\sum }}x_{i} \end{array}$$

(8) $$\begin{array}{l} SD_{rs}=\sqrt{\frac{1}{N-1}\overset{N-1}{\underset{i\;=\;1}{\sum }}{\left(x_{i}-{\bar{X}}_{rs}\right)}^{2}} \end{array}$$

(9) $$\begin{array}{l} CV_{rs}=\frac{SD_{rs}}{{\bar{X}}_{rs}}\times 100\% \end{array}$$

(10) $$\begin{array}{l} E_{rs}=\frac{\left({\bar{X}}_{rs}-T\right)}{T}\times 100\% \end{array}$$

where *N* is the number of rows in the sample plot, *x*_*i*_ is the measured row spacing between two adjacent row lines, ${\bar{X}}_{rs}$ is the mean of the measured row spacings in the sample plot, *SD*_*rs*_ is the standard deviation of the row spacing in the sample plot, and *T* is the theoretical row spacing set by the seeding devices.

The coefficient of variation of seedling uniformity (*CV*_*su*_) among the row lines depended on the rapeseed seedling stand count estimation and row line extraction. As mentioned, the shaking seeding device would lead to irregular seed placement and thus irregular seedling distribution. Therefore, *CV*_*su*_ was calculated using the estimated number of rapeseed objects within 8.0 cm of the row lines:

(11) $$\begin{array}{l} {\bar{X}}_{su}=\frac{1}{N}\overset{N}{\underset{i\;=\;1}{\sum }}u_{i} \end{array}$$

(12) $$\begin{array}{l} SD_{su}=\sqrt{\frac{1}{N}\overset{N}{\underset{i\;=\;1}{\sum }}{\left(u_{i}-{\bar{X}}_{su}\right)}^{2}} \end{array}$$

(13) $$\begin{array}{l} CV_{su}=\frac{SD_{su}}{{\bar{X}}_{su}}\times 100\% \end{array}$$

where *N* is the number of row lines in the sample plot, *u*_*i*_ is the number of rapeseed seedling stand count in the *ith* row line, ${\bar{X}}_{su}$ is the mean of the numbers of rapeseed seedling stand count in the sample plot, and *SD*_*su*_ is the standard deviation of numbers of rapeseed seedling stand count in the sample plot.

## Results

### Object segmentation performance

Table 5 presents the accuracy assessment results using two randomly selected sample plots for the two datasets. In an ideal case that the segmentation is identical to the reference, both Precision and Recall would achieve the maximum value of 1. When an image is under-segmented, the Recall is high but the Precision decreases. By contrast, in an over-segmented situation, the Precision is high but the Recall would decrease. Table 5 shows that the over-segmented situation mainly existed in both ExG and NGRDI over the two stages. All of their Precision values were lower than 80.00%, even 59.23% for NGRDI in Stage 2. On the contrary, most of the Precision and Recall for ExG-ExR and GLI were over 90.00%. Accordingly, ExG-ExR and GLI had greater F-measure, overall accuracy and Kappa value than ExG and NGRDI, indicating their better segmentation performance.

**Table 5:** Segmentation accuracy for two datasets based on four vegetation indices.

**Dataset**	**Vegetation index**	**Precision (%)**	**Recall (%)**	***F*-measure(%)**	**Overall accuracy (%)**	**Kappa value**
**Sample 3 from Stage 1**	ExG	79.25	99.55	89.40	97.69	0.86
	ExG-ExR	93.98	96.63	95.30	99.29	0.96
	GLI	98.66	85.91	92.28	98.09	0.90
	NGRDI	68.57	99.98	84.28	96.57	0.79
**Sample 8 from Stage 2**	ExG	71.52	99.68	85.60	95.28	0.80
	ExG-ExR	83.49	95.72	89.60	96.67	0.87
	GLI	90.85	91.91	91.38	97.18	0.89
	NGRDI	59.23	99.87	79.55	93.28	0.70

The average F-measure, overall accuracy and kappa value for ExG-ExR were 92.45, 97.98 and 0.92%, respectively, compared with the respective values of 91.83, 97.64 and 0.90% for GLI. In fact, ExG-ExR showed an over-segmented in Stage 2, while GLI showed an under-segmented in Stage 1. Supported by the results, ExG-ExR showed better performance than GLI for the segmentation. The results of overall accuracy and Kappa value presented a small difference in segmentation between ExG-ExR and GLI. In contrast, more information about the segmentation performance could be obtained using Precision, Recall and F-measure.

Since ExG-ExR performed slightly better than GLI, it was considered as the best VI for the rapeseed object identification and segmentation. This result illustrated that ExG-ExR combined with typical Otsu thresholding method could be effective for rapeseed object segmentation.

### Rapeseed seedling stand count modeling based on morphological parameters

Table 6 presents the correlation coefficient matrices among the ground-measured rapeseed seedling stand count in segmented objects *(y)* and the 15 shape features (*F*_1_–*F*_15_) for Stage 1 (above the main diagonal) and Stage 2 (below the main diagonal). As shown in Table 6, the absolute correlation values between the ground-measured rapeseed seedling stand count contained in objects (*y*) and the 15 shape features (*F*_1_-*F*_15_) ranged from 0.52 for *F*_12_ to 0.93 for *F*_1_ in Stage 1 and from 0.48 for *F*_12_ to 0.94 for *F*_1_ in Stage 2. These r-values indicated that it was feasible to estimate the rapeseed seedling stand count from the shape features. In addition, the negative correlation coefficient values in both datasets were symmetric about the main diagonal, indicating the consistent negative relationships between these variables.

**Table 6:** Correlation coefficient matrices among ground-measured seedling counting and 15 shape features for Stage 1 (above diagonal) and Stage 2 (below diagonal).

	***y***	***F*_1_**	***F*_2_**	***F*_3_**	***F*_4_**	***F*_5_**	***F*_6_**	***F*_7_**	***F*_8_**	***F*_9_**	***F*_10_**	***F*_11_**	***F*_12_**	***F*_13_**	***F*_14_**	***F*_15_**
y	–	0.93	0.89	0.87	0.70	0.84	0.88	0.71	0.71	0.86	0.70	−0.62	−0.52	−0.69	0.63	0.91
*F*_1_	0.94	–	0.95	0.99	0.68	0.90	0.96	0.79	0.79	0.90	0.71	−0.66	−0.41	−0.75	0.68	0.99
*F*_2_	0.91	0.94	–	0.92	0.66	0.84	0.99	0.68	0.70	0.86	0.65	−0.60	−0.48	−0.66	0.57	0.98
*F*_3_	0.89	0.99	0.89	–	0.60	0.90	0.93	0.80	0.80	0.89	0.65	−0.66	−0.33	−0.76	0.66	0.97
*F*_4_	0.66	0.66	0.64	0.57	–	0.63	0.66	0.57	0.50	0.71	0.89	−0.47	−0.67	−0.55	0.76	0.70
*F*_5_	0.83	0.90	0.78	0.91	0.58	–	0.82	0.91	0.90	0.99	0.68	−0.79	−0.54	−0.87	0.70	0.91
*F*_6_	0.91	0.95	0.99	0.91	0.65	0.78	–	0.68	0.67	0.84	0.64	−0.57	−0.42	−0.64	0.57	0.98
*F*_7_	0.65	0.74	0.56	0.77	0.52	0.89	0.57	–	0.87	0.89	0.64	−0.79	−0.50	−0.94	0.75	0.78
*F*_8_	0.70	0.77	0.62	0.80	0.45	0.90	0.61	0.87	–	0.88	0.59	−0.85	−0.46	−0.89	0.67	0.77
*F*_9_	0.85	0.91	0.82	0.91	0.67	0.99	0.81	0.87	0.88	–	0.76	−0.77	−0.63	−0.86	0.74	0.92
*F*_10_	0.63	0.65	0.60	0.58	0.89	0.61	0.60	0.59	0.52	0.70	–	−0.49	−0.72	−0.59	0.89	0.71
*F*_11_	−0.66	−0.71	−0.59	−0.74	−0.46	−0.87	−0.57	−0.87	−0.93	−0.85	−0.46	–	0.50	0.84	−0.56	−0.66
*F*_12_	−0.48	−0.42	−0.47	−0.35	−0.66	−0.52	−0.43	−0.48	−0.46	−0.61	−0.73	0.53	–	0.54	−0.60	−0.47
*F*_13_	−0.65	−0.73	−0.57	−0.76	−0.49	−0.88	−0.57	−0.94	−0.90	−0.86	−0.53	0.92	0.52	–	−0.71	−0.74
*F*_14_	0.54	0.59	0.47	0.59	0.72	0.65	0.47	0.73	0.63	0.69	0.87	−0.56	−0.60	−0.68	–	0.65
*F*_15_	0.93	0.99	0.97	0.96	0.68	0.89	0.97	0.70	0.73	0.92	0.65	−0.68	−0.48	−0.69	0.56	–

*All r-values were significant at the 0.01 level (double tail)*.

*
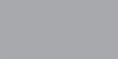
Indicated Stage 1 (above diagonal)*.

*
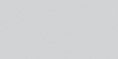
Indicated Stage 2 (below diagonal)*.

Through the pre-selection process, the same three significant shape features (*F*_4_, *F*_12_, and *F*_15_) were chosen for both two stages. None of the three features were removed during the multiple stepwise regression modeling. Table 7 gives the regression analysis results based on the three shape features. The three shape features (Length-width Ratio, Density, Border length) were significant at the 0.05 level with *R*^2^-values of 0.845 for Stage 1 and 0.867 for Stage 2. Although Stage 2 had a slightly higher *R*^2^-value, it also had a slightly higher RMSE. A single model was also fitted over the two stages in this study. The single model showed a good agreement with an R-square of 0.846. The total number of rapeseed plants could be accurately estimated with an average relative error of 6.83%. The result also demonstrated that it was feasible to estimate the number of rapeseed plants with shape traits of segmented objects during these two stages. However, the similar results from the two individual stages, which were 10 days apart, and from the combined stage indicate that there was no significant difference between the two stages as far as image acquisition timing is concerned. Clearly, it is not possible to determine an optimal time window from only two stages. A multi-stage experiment is necessary to find out the optimal time window in future research. Nevertheless, the two stages used in this study were within the reasonable time window based on the results.

**Table 7:** Regression models for Stages 1 and 2 and for the two stages combined.

**#**	**Model**	***F*-value**	***R*^2^**	**RMSE**	***P*-value**
Model A from Stage 1	*y* = 0.382*F*_4_-1.188*F*_12_ + 0.011*F*_15_ + 2.364	3863.525	0.845	0.672	<0.05
Model B from Stage 2	*y* = 0.167*F*_4_-0.621*F*_12_ + 0.009*F*_15_ + 1.498	5874.029	0.867	0.759	<0.05
A single model	*y* = 0.462*F*_4_-0.747*F*_12_ + 0.009*F*_15_ + 1.465	8863.680	0.846	0.747	<0.05

*y is the estimated rapeseed seedling stand count of per segmentation object*.

A comparison between estimated numbers of plants per object and measurements among the sample plots showed that the distribution of the numbers of plants per object was positively right-skewed. The numbers of plants per object ranged mostly from 1 to 3 based on ground observations, and the estimated average numbers of plants per object also ranged from 1 to 3. Since the rapeseed plants in this study were mainly at early growth stages the size and overlap of their leaves were relatively small. However, the number of plants per object can be much larger, indicating the complexity of the plant estimation from it. Nevertheless, the results demonstrated that their morphological traits contained detailed information of the seedling stand count.

Table 8 gives the statistical results of each model for the different validation datasets. The two models produced high *R*^2^-values with RMSE values less than 1, indicating good estimation accuracy. Moreover, the MAE values of the two models were <10% with only about 5% for Model B. The sub-dataset for PPCCSD (Sample 5 in Model A and Sample 6 in Model B) had the lowest *R*^2^-values and, highest RMSE and *E*_s_ among the validation datasets. Validations A and B generally showed better performance than most of the validation datasets representing the three seeding devices.

**Table 8:** Validation results of two models.

**Model**	**Sub-dataset**	**Number of objects**	***R*^2^**	**RMSE**	**Measured sum**	**Estimated sum^*a*^**	***E_*s*_* (%)**	**MAE (%)**
A	Validation A	376	0.862	0.72	694	721	3.89	9.79
	Sample 5 (PPCCSD)	83	0.718	0.85	147	177	20.41	
	Sample 10 (RDSD)	221	0.794	0.63	319	362	13.48	
	Sample 17 (CMD)	234	0.889	0.62	431	425	−1.39	
B	Validation B	477	0.886	0.72	846	859	1.54	5.11
	Sample 6 (PPCCSD)	133	0.846	0.88	255	281	10.20	
	Sample 8 (RDSD)	197	0.886	0.58	459	442	−3.70	
	Sample 13 (CMD)	242	0.858	0.61	440	418	−5.00	

a *Estimated sum was rounded to integers*.

### Rapeseed row characteristics for seeding performance evaluation

**Figure 5:**
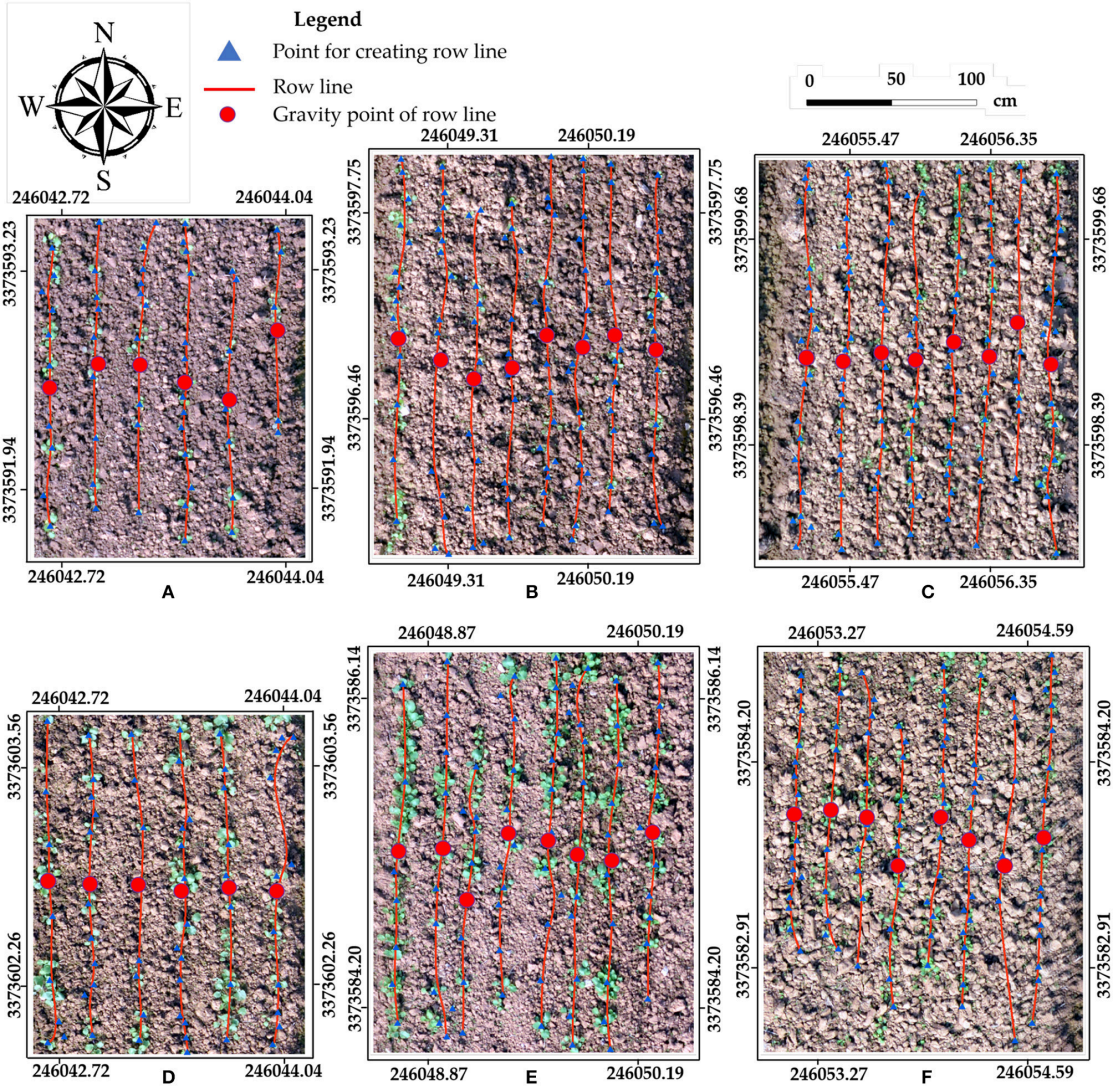
Row line extraction using six samples for three seeding devices in two datasets. For Stage 1: Sample 5 (PPCCSD) **(A)**, Sample 10 (RDSD) **(B)**, and Sample 17 (CMD) **(C)**. For Stage 2: Sample 6 (PPCCSD) **(D)**, Sample 8 (RDSD) **(E)**, and Sample 13 (CMD) **(F)**. Samples 5 **(A)** and **(B)** had smaller sampling plots.

The rapeseed row line extraction results for the six sample plots are displayed in Figure 5. The row line extraction was conducted on the validation datasets representing the three different seeding devices. The red row lines were created by connecting the blue triangle points (Points for Lines Creating in Figure 4). The smoothed line processing in Figure 4 should be noted. It had little impact on the CV of row spacing and the row spacing error. The average CV of row spacing for non-smoothed was 10.80%, and the average row spacing error was 10.66%. After smoothed processing, the average CV of row spacing slight degraded to 9.89%, while the average row spacing error increased to 10.74%. In fact, the variation of the seeding devices would lead to irregular seed placement and thus irregular seedling distribution. In practical seeding operations, there was a tolerated distance. On the other hand, through the smoothed processing, the extracted line would be similar to the central line of the row, which would approximate the real row line.

In Figure 5A, the row line on the east edge of the sample plot was shorter than the other row lines due to missing rapeseed seedling stand, and the same also occurred in Figure 5F. Furthermore, the row lines had some distortion. The row lines were created by connecting the points transformed from the segmentation objects. The missing rapeseed plants would increase the distance between the two major points in the same rapeseed row. If these two points had some obvious horizontal offset, or if a point deviated from the row line was located between the two major points, the connected row line would not be straight due to the change of direction. Moreover, the randomness and subjectivity of the sample plot delimitation also influenced the results. Although there were some deficiencies, using geographic coordinate information of these points to create row lines was appropriate. More importantly, this method could obtain the geographic coordinate information for seeding performance evaluation.

**Figure 6:**
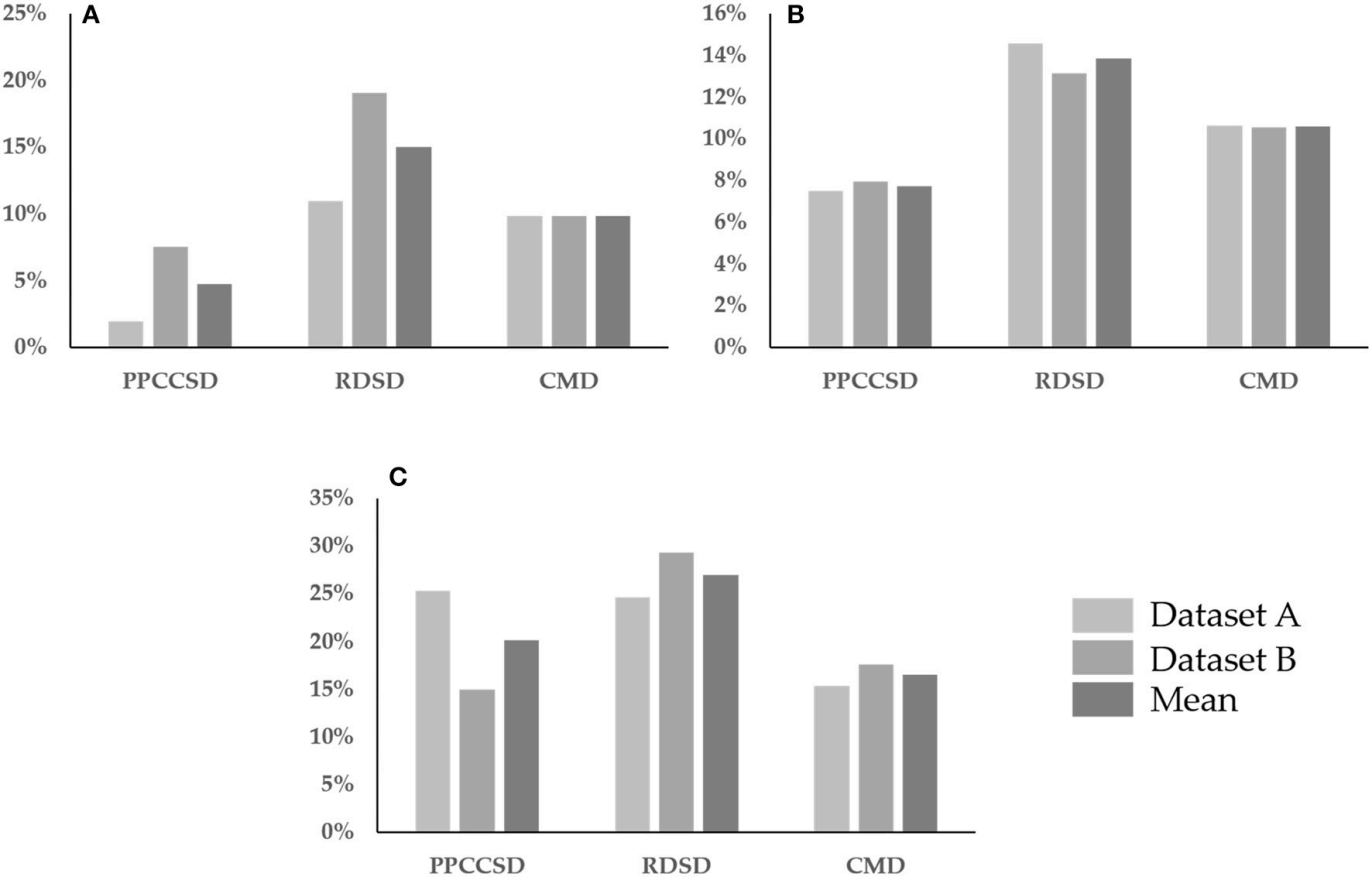
Comparison of three row characteristics for three seeding devices, the CV of row spacing uniformity in **(A)**, the row spacing error in **(B)** and the CV of seedling uniformity in **(C)**. Three seeding devices are precision pneumatic cylinder-type centralized seeding device (PPCCSD), rotating disc-type seeding device (RDSD), centrifugal metering device (CMD).

Figure 6 shows three row characteristics for the three seeding devices for the two observation dates. The values for the three row characteristics were higher for Stage 2 than for Stage 1 except for the row spacing error in RDSD for Stage 1 (Figure 6B) and the CV of seedling uniformity in PPCCSD for Stage 2 (Figure 6C). These row characteristics were helpful to recognize corresponding seeding abnormal areas for seeding performance evaluation.

The higher CV of seedling uniformity in PPCCSD for Stage 1 as shown in Figure 6C was partly due to the row line on the east edge of the sample plot was shorter than the other lines because of the missing rapeseed stand as shown in Figure 5A. The accuracy of seedling stand count estimation was another factor for the higher CV value. The estimation for this dataset (Sample 5) had the lowest *R*^2^ of 0.718 as shown in Table 8. The three row characteristics for CMD had less fluctuation than those for the other two seeding devices. The variation of growth condition between Samples 17 and 13 for CMD was the least among the three seeding devices (Figure 5), explaining why the row characteristics of CMD had a relatively less fluctuation. Sample 13 contained four row lines shorter than the other four lines because of poor rapeseed stand, which may have caused the higher CV of seedling uniformity as shown in Figure 6C.

Multiple factors can also affect the seeding performance. Variability in soil texture and microtopography can result in differences in emergence and growth. Field variability might be the reason for the higher row spacing error in RDSD (Figure 6B). The obvious shadows on the bare soil background as shown in Figure 5B were due to the coarseness of soil texture and variation of microtopography.

In summary, it was practicable to evaluate seeding performance through the seedling stand count based on an UAV field-based HTPP. Seeding abnormal areas could be recognized by the crop row characteristics. These row characteristics were helpful for evaluating the rapeseed emergence and growth conditions, and the seeding performance evaluation was useful for effective crop management within the season. In addition, remote sensing images can be collected shortly before or after seeding to document pre-emergence field conditions for seeding performance evaluation.

## Discussion

### Limitation of spectral information

**Figure 7:**
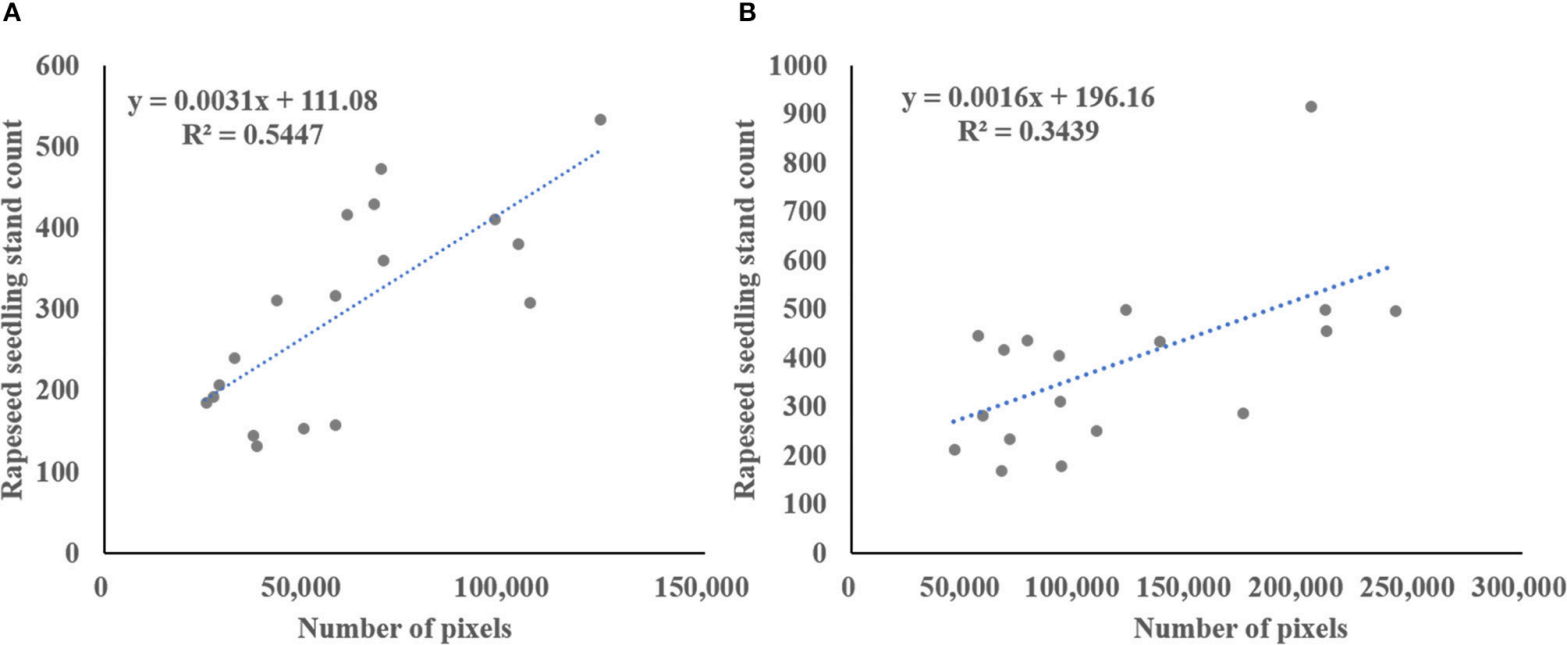
Correlation between ground-based rapeseed seedling count and the number of seedling pixels extracted from ExG-ExR in the sample plots for Stage 1 in **(A)**, and Stage 2 in **(B)**.

The spectral information in the visible bands was used for rapeseed object identification and segmentation in this study. Consumer-grade RGB camera was used for image acquisition. The usefulness of this type of cameras for crop identification has been demonstrated (Zhang et al., 2016, 2017). The color VIs derived from only RGB spectral bands can accentuate a color that may be intuitive for comparison of plant greenness (Meyer and Neto, 2008). Color VIs are suggested to be less sensitive to lighting variations (Meyer and Neto, 2008; Campbell and Wynne, 2011), but impact of sunlight conditions is unavoidable. Compared to indoor experiments, image acquisition in field-based experiments is more difficult. It is impossible to control the sunlight outdoors, but imaging can be carried out under relatively sunny conditions. In this study, the weather of during image acquisition was clear and calm to minimize the impact of sunlight change. Therefore, the segmentation results revealed that almost all the rapeseed objects were successfully identified and separated from the background with satisfied accuracy (Table 5). Color VIs combined with typical Otsu thresholding method were effective for rapeseed object identification and segmentation. However, using spectral information alone for data analysis limited the quantitative interpretation of vegetation remote sensing information (Xue and Su, 2017; Yang et al., 2017). For instance, it was a challenge for seedlings stand counting due to complex overlapping of rapeseed seedlings. Figure 7 illustrates the relationship between total ground measured rapeseed seedling stand count and number of seedling pixels extracted from ExG-ExR for the two datasets. These results indicated that the use of spectral information alone could not sufficiently estimate rapeseed seedling stand count in the sample plots compared with the models presented in Table 7. As rapeseed seedlings grew with more leaf overlapping, the correlation would further decrease.

Previous studies have demonstrated that RGB imagery has the capability to detect and count post-emergence plants more easily at their growth stages for cotton (Chen et al., 2018), maize (Gnädinger and Schmidhalter, 2017), and potato (Sankaran et al., 2017). Most of these plants were represented by individual objects after identification and segmentation using spectral information, because they were bigger with larger spacing and more uniform distribution (Jin et al., 2017; Liu et al., 2017b). Moreover, the Near infrared (NIR) band has shown its spectral sensitivity for crop vegetation detection (Zhang et al., 2016). The accuracy may be improved with additional NIR images (Chen et al., 2018). High resolution multispectral images were successfully used to estimate crop emergence in potatoes (Sankaran et al., 2017). These results demonstrated that spectral information could be effective to identify and count the number of seedlings with no or minimal overlapping. However, it was difficult and in feasible to estimate plant count if complex plant overlapping occurs as the unsatisfying results shown in Figure 7.

The spectral information was still necessary for the identification and segmentation for such complex overlapping crop as rapeseed. With complex overlapping, each segmentation objects does not necessarily represent an individual plant (Jin et al., 2017; Liu et al., 2017b). Accordingly, additional information is needed to supplement the spectral information. For example, a skeleton analysis of the touching wheat seedlings was used to count the wheat seedlings after segmentation (Liu et al., 2016). Compared to wheat, rapeseed has elliptic leaves, so the skeleton analysis was hard to apply. Therefore, other features such as morphological features were needed to count the number of rapeseed seedlings.

### Importance of morphological parameters

The OBIA technique was conducive to obtain more features of the segmentation objects for the rapeseed seedling stand counting. Size and shape analysis of corn plant canopies demonstrated that morphological information was useful for plant population and spacing sensing (Shrestha and Steward, 2005). Although texture and morphological characteristics were used to count wheat seedlings (Cointault and Chopinet, 2006; Jin et al., 2017; Liu et al., 2017b), but little work has been done for rapeseed.

This study extracted 15 morphological features and confirmed the morphological features could be used for estimating rapeseed seedling stand count (Table 6). Repeated random experiments to verify the lower *r*-values for *F*_12_ showed that the average absolute correlation coefficient value between *y* and *F*_12_ was about 0.5 in both datasets. Although *F*_12_ had the lowest correlation with *y*, it was a significant variable in the multiple regression models between the number of rapeseed seedlings per object and the three shape features. Moreover, it was important to compare the models for the two datasets and to even obtain a universal model for estimating rapeseed seedling stand count based on the three selected shape features (*F*_4_, *F*_12_, and *F*_15_). *F*_4_ was length-to-width ratio of an image object's minimum enclosing rectangle. *F*_12_ was the distribution in space of the pixels of an image object. *F*_15_ was the sum of pixels along an image object edge (Table 4). Although these three shape features had different coefficients, they had the same positive or negative sign in both models (Table 7). As shown in Table 6, *F*_4_ and *F*_15_ had a positive correlation, and *F*_12_ had negative correlations with *F*_4_ and *F*_15_. It can be clearly seen from the models that the estimated number of the rapeseed seedlings in a segmentation object increases with *F*_4_ and *F*_15_ and decreases with *F*_12_.

The results presented in Tables 7, 8 indicated that using multiple regression to establish the rapeseed seedling stand count models was appropriate and feasible. The use of morphological parameters significantly improved the accuracy for rapeseed seedling stand count estimation compared with the use of spectral information.

### Importance of image acquisition time

Crop growth is a dynamic process (Sankaran et al., 2017; Chen et al., 2018). Image acquisition time can affect crop monitoring and analysis results (Liu et al., 2017c). Compared to some wheat seedling counting studies based on single dates (Jin et al., 2017; Liu et al., 2017b), this study employed the images captured at two different growth stages of the rapeseed crop.

The growth condition initially influenced the performance of segmentation (Table 5). The differences in canopy coverage might have resulted in the different superior VIs for the datasets. Ground canopy cover was considered as an important trait related to crop growth (Mullan and Reynolds, 2010). As crop plants grow, canopy cover will become saturated. The experiments in this study were carried out at two early growth stages. There were only 10 days apart between these two stages. Despite the short time interval, our calculations showed that the average canopy cover increased about 50% between the two stages among all sample plots. There are significant differences over these two stages, representing the rapid change of rapeseed plant growth. Nevertheless, more observations are still necessary to examine the effect of image acquisition time in future work.

Crop growth conditions also impacted the performance of the seedling count models (Tables 7, 8 and Figure 7). There were differences in emergence of the rapeseed seedlings at the two growth stages (Table 8 and Figure 5). By examining the modeling data, the maximum seedling stand count contained in segmented objects in the two datasets was 16 for Stage 1 and 25 for Stage 2. The most probable reason for the lower seedling count for the first date was due to the missing and extremely small rapeseed seedlings from delayed germination. If the crop emergence is completed, all emerged seedlings and the region of missing seedlings would be more obvious for evaluation. Compared with the acquisition time of Stage 1, the time of Stage 2 was therefore more suitable in this study. Observation time was a crucial factor for seeding performance evaluation (Liu et al., 2017c). Furthermore, weeds and growing rapeseed seedlings could be distinguished more easily on the second date because their differences in leaf area and color were more obvious.

The single model covering the two stages would inspire the further improvement of this research. Multiple observations should be made during the critical crop growth stages in the future. With multiple observations, the seedling stand count models derived from the data can be more accurate and reliable. Moreover, the same sample plot for each seeding device can be observed multiple times for its spatial-temporal change for seeding performance evaluation. The temporal change of the extracted row characteristics will provide more information on the emergence and crop growth. Thus, the best acquisition time for rapeseed seedling stand count and seeding performance evaluation can be determined.

## Conclusions

As low-attitude UAV remote sensing technology is being increasingly used for monitoring agricultural fields, this study developed practical methods for estimating directly-seeded rapeseed seedling stand count and for evaluating seeding performance using ultra-high resolution RGB images captured by the low-altitude UAV remote sensing platform.

Color VIs combined with Otsu thresholding method were efficient and reliable for rapeseed seedling object identification and segmentation. The result showed that ExG-ExR and GLI performed better than ExG and NGRDI. Meanwhile, ExG-ExR was a bit better than GLI supported by the data in this study. Multiple regression analysis was used for seedling count modeling with extracted shape features. Two models for each stage showed good agreement between the seedling stand count with three shape features (Length-width Ratio, Density, Border length). A single model over these two stages reaffirmed the feasibility to estimate the number of rapeseed plants with shape traits of segmented objects. These results clearly showed that there existed significant relations between the number of directly-seeded rapeseed seedlings in a segmentation object and its shape features, indicating traditional multiple regression analysis was a rapid and effective method for modeling rapeseed seedling stand count. However, the results also revealed that further work should pay more attention to multiple and long-term observations.

This study also illustrated an application to seeding performance evaluation based on the rapeseed seedling identification and stand count estimation. Rapeseed plant rows were extracted from the georeferenced segmentation objects. Results from the sample datasets showed that the object spatial information was sufficient to perform crop row extraction, though there were some limitations due to the uncertainty in sample delimitation and the process for point sorting and categorizing. The seeding performance evaluation using three row characteristics (row spacing error, CV of row spacing and CV of seeding uniformity) showed that it was feasible to use the information derived from low-attitude UAV image data for evaluating the performance of mechanical seeding devices.

UAV-based remote sensing has great potential for field-based phenotyping with the advances of this technology. Future work can be focused on the spatial-temporal variation during the rapeseed growing season. Meanwhile, it is necessary to develop a generalized model for estimating rapeseed seedling count and to use the model for improving the performance of mechanical seeding devices and optimizing the efficiency of rapeseed production.

## Author contributions

JZ and BZ designed and conducted the remote sensing part of the experiment. QL and YD designed and conducted the agronomy part of the experiment. JZ, BZ, and JX processed and analyzed the imagery as well as wrote the manuscript. CY guided the study design, advised in data analysis, and revised the manuscript. GZ, YS, and DZ were involved in the process of the experiment, ground data collection, or manuscript revision. All authors reviewed and approved the final manuscript.

### Conflict of interest statement

The authors declare that the research was conducted in the absence of any commercial or financial relationships that could be construed as a potential conflict of interest.

## Abbreviations

UAVs: unmanned aerial vehicles
FBP: field-based phenotyping
HTPPs: high-throughput phenotyping platforms
VIs: vegetation indices
PPCCSD: precision pneumatic cylinder-type centralized seeding device
RDSD: rotating disc-type seeding device
CMD: centrifugal metering device
ExG: excess green index
ExR: excess red index
ExG-ExR: excess green minus excess red index
NGRDI: normalized green minus red difference index
GLI: green leaf index.
